# Evaluation of functioning and associated factors in children and adolescents with osteogenesis imperfecta

**DOI:** 10.1590/1984-0462/2025/43/2023193

**Published:** 2024-09-09

**Authors:** Arthur Cherem Netto Fernandes, Têmis Maria Félix

**Affiliations:** a Universidade Federal do Rio Grande do Sul, Porto Alegre, RS, Brazil.; b Hospital de Clinicas de Porto Alegre, Porto Alegre, RS, Brazil.

**Keywords:** Osteogenesis imperfecta, International classification of functioning, Disability and health, Child development, Rehabilitation, Osteogênese imperfeita, Classificação internacional de funcionalidade, Incapacidade e saúde, Desenvolvimento infantil, Reabilitação

## Abstract

**Objective:**

The aim of this study was to evaluate the functioning and associated factors in children and adolescents with osteogenesis imperfecta (OI).

**Methods:**

This is a cross-sectional study conducted on 30 children and adolescents with OI. Medical records, use of bisphosphonates, socioeconomic status, handgrip strength, balance, joint hypermobility, ambulatory level, and the Pediatric Evaluation of Disability Inventory—Computer Adaptative Test (PEDI-CAT) scores were assessed. Data is presented as mean and standard deviation and Student’s t-test or Mann–Whitney U test. Categorical data is presented as frequency and analyzed using Fisher’s exact test. Within-group analyses were conducted using ANCOVA or Wilcoxon signed-rank test. Correlations used Kendall’s Tau-b test.

**Results:**

The participants involved in this study were 6–18 years old. The sample was separated into two groups according to disease severity. The moderate/severe OI group (n=10) presented a lower height and muscular strength than the mild group (n=20). Muscle weakness was observed in all participants with OI when compared with the normal population. No differences were observed between the groups in the PEDI-CAT scores except for the mobility domain. There were correlations between the PEDI-CAT mobility domain and the number of fractures, OI type, weight, and balance; there was also a correlation between the PEDI-CAT daily activities, mobility, responsibility, and social/cognitive domains.

**Conclusions:**

The findings suggest that children with moderate/severe forms of OI can achieve the same function levels as children with mild OI. Fractures can have a major influence on the functional level, and treatment should focus on the prevention and rehabilitation of these events when they occur.

## RESUMO

**Objetivo:**

Avaliar a funcionalidade e fatores associados em crianças e adolescentes com osteogênese imperfeita (OI).

**Métodos:**

Estudo transversal com 30 crianças e adolescentes com OI. Foram avaliados prontuários médicos, uso de bisfosfonatos, características socioeconômicas, dinamometria de preensão palmar, equilíbrio, hipermobilidade articular, nível de deambulação e escores do *Pediatric Evaluation of Disability Inventory - Computer Adaptative Test* (PEDI-CAT). Os dados foram apresentados em média e desvio padrão e comparados por teste *t* por Mann-Whitney, enquanto os categóricos foram apresentados em frequência e comparados pelo teste exato de Fisher. Análises intragrupos foram realizadas por análise de covariância (ANCOVA) ou Teste de Wilcoxon para postos sinalizados. O teste Tau-b de Kendall foi usado para correlações.

**Resultados:**

A idade variou de 6 a 18 anos. A amostra foi dividida em dois grupos de acordo com a gravidade da doença. Casos moderados/graves (n=10) apresentaram menor estatura e força muscular comparadas às dos leves (n=20). Fraqueza muscular foi observada em todos os casos de OI quando comparados à população normal. Não houve diferença nos domínios do PEDI-CAT com exceção do domínio mobilidade. Houve correlação entre o número de fraturas, tipo de OI, peso e equilíbrio e o domínio mobilidade; e entre os domínios Atividades Diárias e Mobilidade e Responsabilidade e Social/cognitivo do PEDI-CAT.

**Conclusões:**

Nossos achados sugerem que crianças com OI moderada/severa podem atingir o mesmo nível de funcionalidade que crianças com a forma leve. Fraturas podem ter grande influência no nível de funcionalidade e o tratamento deve enfocar a prevenção e a reabilitação desses eventos.

## INTRODUCTION

Osteogenesis imperfecta (OI) is a rare genetic disorder—with a prevalence of 1 in 10,000 to 20,000 live births — that leads to bone fragility due to a collagen type I biosynthesis defect. The disorder is classified into various subtypes based on clinical and radiological criteria, including type I (mild), type II (perinatally lethal), type III (severe and progressive deforming), type IV (moderate), and type V (moderate with specific findings). Type V includes interosseous membrane calcification, displacement of the radial head, and hyperplastic callus.^1^ The high risk of fractures and chronic pain associated with OI can lead to severe restrictions in daily activities and can require pharmacological treatment and physiotherapy. The clinical characteristics of OI can negatively impact health-related quality of life. The degree of the impact is often associated with the severity of the disease.^2,3^

Muscle strength and bone deformities are factors that affect children with OI and lead to impaired mobility.^4^ Bisphosphonate administration may influence the functioning and pain levels of children with OI.^4^ Physical activity needs to be maximized without leading to fractures in children with OI because it is a form of social participation and preventive therapy for obesity and cardiovascular disease, which are common, especially in severe forms of OI.^5^ The perceived competence of children with OI is also reduced in several aspects of life.^6^ Treatment must focus on mobility, independence, and self-care abilities.^7^

This study aimed to evaluate the functioning of children and adolescents with OI considering associated factors such as the use of bisphosphonates and physical activity and to compare children with mild OI and children with moderate/severe OI.

## METHOD

This cross-sectional study was conducted at the Reference Center on Osteogenesis Imperfecta at the Clinical Hospital of Porto Alegre (CROI-HCPA). The inclusion criteria were a clinical diagnosis of children with OI who aged between 6 and 19 years. The exclusion criteria were scores higher than 5 on the Visual Analog Pain Scale and fractures within the last 4 months (as this could influence the functional abilities of the children). This study was approved by the Ethics and Research Committee at the Clinical Hospital of Porto Alegre (CAAE: 15257519.0.0000.5327). The patients were recruited on the same day they had a clinical appointment with a physician. The participants and/or their legal guardians signed an informed consent form before participation. All the measures were exclusively done by one physical therapist.

The evaluation consisted of a clinical and structured questionnaire with questions on demographics, treatments, the date of the last fracture, the total number of fractures, previous surgeries, the ambulation level, physical activity, and physiotherapy during the care process. Socioeconomic data were collected using the Brazilian Association of Research Companies Economic Classification Criterion (ABEP), which labels family income from A1 (the best classification) to E (the worst classification). The criteria take into consideration material items present in the family home, the level of schooling of the head of the family, and the infrastructure present.^3^ Higher socioeconomic levels can provide an advantage in the motor development of children, especially younger children.^8^ Bone mineral density (BMD) and use of bisphosphonates were collected from medical records and are expressed as z-scores.

The children and parents were asked about physical activity practice, including any sporting or ludic activities performed weekly. They were also asked about receiving physiotherapy; this was considered a dichotomous variable (yes or no).

Ambulation level was evaluated because it is related to body functions and structures such as muscular strength, the number of fractures, and the presence of deformities.^9^ The Bleck scale considers five levels of ambulation: non-ambulation (0), therapeutic walking (1), household walking (2), community walking (3), and independent walking (4).^10^

Muscular strength was evaluated using handgrip dynamometry (JAMAR Hydraulic Hand Dynamometer 5030J1, Chicago, IL, USA). Three measurements were performed by using the dominant hand with at least a 1-min interval between each test. The highest value was recorded and is expressed in kilogram-force (kgf). Muscular strength is often reduced in patients with OI and may cause impairments in mobility and motor development.^6,9,11^ The results were compared according to age and sex with data from the standard Brazilian population of the same age.^12^

The Pediatric Balance Scale (PBS) involves 14 tasks that assess static and dynamic balance with progressive levels of difficulty, starting with “standing up from a chair” and “finishing with leaning the body forward without moving the legs.” The overall score can be as high as 56 points, with higher scores indicating better balance.^13^ The PBS was administered to all children who could stand up without help. The PBS was chosen because it correlates with tools that evaluate functioning, such as the Gross Motor Function Measure (GMFM) and the Pediatric Evaluation Disability Inventory (PEDI).^13^

Joint hypermobility was evaluated because it is present in most children and could affect motor development. Hypermobility was assessed based on the Beighton criteria, which assigns four bilateral points for thumb, little finger, knee, and elbow hyperextension, and one point for lumbar column hyperflexion. Hypermobility is present if the individual scores ≥5 points.^9^

Functioning was measured using the validated Brazilian version of the Pediatric Evaluation of Disability Inventory — Computer Adaptative Test (PEDI-CAT).^14^ The PEDI-CAT measures abilities in four domains: daily activities, mobility, social/cognitive, and responsibility. The daily activities domain evaluates self-care and domestic activities, the mobility domain evaluates the ability of the individual to move in diverse terrains, the social/cognitive domain evaluates interactions inside and outside the familial environment and the capacity to sustain relationships, and the responsibility domain evaluates how much the child/adolescent assumes responsibility for a specific activity and how much their caregivers assume. All the questions were answered by the mother, except for one 18-year-old participant who came alone to the appointment. We used the balanced version of the PEDI-CAT with 30 items per domain, selected from a 256-question repository.^14,15^ There are five possible answers to each question that represent the degree of assistance required for each activity. The results are expressed as a continuous score, a T-score, and an age-specific percentile. The mean result for each of the 21 age groups is 50 with a standard deviation of 10. Results between two standard deviations, 30 and 70, are considered within the expected for that specific age according to the manual of the instrument.

The sample size was calculated with the WinPepi software, considering an α value of 0.05, a power of 80%, and previous studies that used the PEDI score. The estimated sample size was 44 children and adolescents.

The results are expressed as the mean or median±standard deviation. The Shapiro-Wilk test was used to determine whether the data were normally distributed. Categorical data were tested using Fisher’s exact test, continuous variables were tested using Student’s *t*-test or the Mann-Whitney U test, and analysis of covariance (ANCOVA) or the Wilcoxon signed-rank test was used for intragroup analyses. For correlation analysis, the Kendall Tau-b test was used due to the small size sample. SPSS Statistics version 25.0 (IBM Corp., Armonk, NY, USA) was used for all analyses. The significance level was set at p<0.05.

## RESULTS

The sample comprised 30 children and adolescents with OI from a pool of 150 families, with 70 children available for screening. All invited subjects agreed to participate in the study. The participants were divided into two groups: mild cases with OI type I (n=20) and moderate/severe cases with OI types III, IV, and V (n=10). The moderate/severe group included eight children with OI type IV and one child each with types III and V. No children were excluded, and all the procedures were performed during a single appointment. The sample characteristics are shown in Table 1. There were significant between-group differences in age (p=0.002), weight z-score (p<0.001), and height z-score (p<0.001), with higher values observed in the mild group.

**Table 1:** Clinical characteristics of the sample according to severity of osteogenesis imperfecta.

	Mild cases (n=20)	Moderate/severe cases (n=10)	p-value
Age (years/SD)	14.10±3.2	10.60±4.6	0.022
Median (min–max)	14 (6 to 18)	9.50 (6 to 19)	
Male (n/%)	13 (65%)	3 (30%)	0.077
Height (z-score)	0.48 (-0.69 to 1.50)	-0.98 (-1.80 to 1.0)	<0.001
Weight (z-score)	0.40 (-0.88 to 2.01)	-0.75 (-1.5 to 0.8)	<0.001
Bisphosphonate (n/%)	11 (55)	7 (70)	0.694
Physical therapy (n/%)	6 (30)	4 (40)	0.440
Physical activity (n/%)	8 (40)	3 (30)	0.702
Hypermobility (n/%)	13 (65)	6 (60)	0.559
Orthopedic surgery (n/%)	15 (75)	9 (90)	0.559
Socioeconomic class*	12.3±3.0	15.2±5.2	0.060
C1	0	1	
C2	2	2	
D/E	18	7	
Bleck scale			0.224
0	1	2	
1	1	0	
2	0	1	
3	2	2	
4	16	5	

Data expressed in mean±standard deviation; median (min–max);

*according to Brazilian Association of Research Companies.

Twenty-one children (70%) presented with full ambulation. There was a positive correlation between the use of bisphosphonates and the Bleck scale classification (r=0.379 and p=0.039), and a negative correlation between the use of bisphosphonates and the number of fractures (r=-0.391 and p=0.009). The average PBS score was 53 points, which is within the normal range of children with typical development (Table 2). The PBS score correlated positively with the Bleck scale (r=0.662, p<0.0001) and negatively with the number of fractures (r=-0.364, p=0.007).

**Table 2:** Evaluation of handgrip strength, bone mineral density, and pediatric balance scale.

	OI total	Mild cases	Moderate/severe cases	p-value (95% CI)
Handgrip strength (kg/f)	14.43±8.95	17.40 (8 to 40)	8.90 (5 to 15)	0.080*
BMD (TBLH) (z-score)	-1.29±1,30	-1.23±1.06	-1.39±1.71	0.775 (-0.925 to 1.228)
BMD (LC) (z-score)	-1.72±1.16	-1.73±1.18	-1.71±1.18	0.957 (-0.964 to 0.914)
PBS (min–max)	52 (20 to 56)	53 (20 to 56)	53 (42 to 56)	0.588

Data expressed in mean ± standard deviation; median (min–max), BMD: Bone Mineral Density; TBLH: Total Body Less Head; LC: Lumbar Column; PBS: Pediatric Balance Scale (n=27);

*p-value between mild cases x moderate/severe cases adjusted by age using analysis of covariance (ANCOVA). BMD differences tested with Student’s *t*-test and PBS with Mann-Whitney U test.

Regarding handgrip strength, there was a significant difference between children with OI (14.57 kgf) and typically developing children (27.79 kgf, p<0.001). This difference remained when both groups were evaluated independently (mild group at 17.40±11.24 kgf and moderate/severe group at 8.90±4.06 kgf) and compared with healthy children. Comparison between the groups showed no difference when age correction was applied to data (Table 2).

For BMD, there was no difference in total body less head (TBLH) and lumbar column (LC, L3–L4) between the groups. Regarding the use of bisphosphonates, there were significant differences between groups in TBLH (p=0.04, 95%CI -1.983 to -0.0291).

PEDI-CAT domains were analyzed using the T-score (Table 3). No participant had a T-score higher than 70. For the daily activities domain, 80% of the mild group and all of the moderate/severe group had a score in the normal range. For the mobility domain, only 35% of the mild group and 80% of the moderate/severe group had a score in the normal range. For the responsibility domain, 97% of the participants had a score in the normal range. Finally, for the social/cognitive domain, 90% of the participants had a score in the normal range.

**Table 3:** Pediatric Evaluation of Disability Inventory-Computer Adaptative Test results.

Domain	T-score	OI-Total (n)	Mild cases (n)	Moderate/severe cases (n)	p-value	r
DA	<30	4	4	0	0.135	0.392^†^
	30–70	26	16	10		
MO	<30	15	13	2	0.02*	
	30–70	15	7	8		
SC	<30	3	3	0	0.204	0.557^‡^
	30–70	27	17	10		
RE	<30	1	1	0	0.480	
	30–70	29	19	10		

DA: Daily Activities; MO: Mobility; SC: Social/Cognitive; RE: Responsibility;

*Mann-Whitney U test;

^†^p-value for Kendal Tau-b correlation of 0.03 between DA and MO;

^‡^p-value for Kendal Tau-b correlation of 0.001 between SC and RE.

The PEDI-CAT results are shown in Table 3. There was a correlation between the mobility domain score and the number of fractures (r=-0.478, p=0.007), weight z-score (r=-0.511, p=0.005), and the PBS score (r=0.422, p=0.02). There was a correlation between the mobility and daily activities domain scores (r=0.392, p=0.03) and between the responsibility and social/cognitive domain scores (r=0.557, p=0.001). Moreover, there was an association between the mobility domain score and OI type (p=0.02).

When comparing children with a score of <30 and 30–70 in the mobility domain, there was a significant difference in the weight z-score (p=0.001, 95%CI 0.467–1.749), number of fractures (p=0.01), and the PBS score (p=0.023). All the differences were favorable to the group with a T-score of 30–70. There were no differences for dynamometry, height z-score, and use of bisphosphonates in any of the PEDI-CAT scores.

## DISCUSSION

The sample shows a predominance of mild OI cases. This is consistent with previous observations indicating that type I could represent 45% of OI cases, the same percentage as types III and IV combined.^16^ The number of fractures was not significantly different between groups. This could be due to the previous use of bisphosphonates, which was reported by 60% (n=18) of the participants. Bisphosphonates are used in the standard treatment for OI and can improve BMD and reduce fractures and pain.^10^ Short stature is one of the main characteristics of OI, especially in the severe forms of the disorder.^17^ Thus, the group differences in height and weight seen in the current study are expected.

Eleven participants (36.67%) reported weekly physical activity. Of these individuals, seven reported the time spent on these activities, and one participant reported more than 300 min of physical activity per week. A recent review identified factors that interfere with adherence to physical activity in patients with OI: Some are intrinsic to the disorder, such as muscular weakness and short stature, while others are related to the fear of fractures or to caregiver/parent overprotection. The lack of physical activity can aggravate muscular weakness and further reduce cardiopulmonary capacity.^18^ Despite the recommendation, only 10 subjects (33%) reported ongoing treatment with a physical therapist. Physical therapy is recommended for rehabilitation after fractures or orthopedic surgery and also to prevent scoliosis, reduced mobility, long-bone deformities, and muscular weakness.^19^ In Brazil, late diagnosis, distance to a health care center, lack of transportation, and shortage of physiotherapists present barriers to OI care.^20^ The majority of the participants in this study (n=19) lived outside the metropolitan area, where the CROI-HCPA is located, and reported a lack of local resources.

Children with OI type I are typically full ambulators or community ambulators without crutches.^21^ Children with OI type IV can achieve a score of 3 or 4 on the Bleck scale, corresponding to community and full ambulation, respectively, especially after bisphosphonate treatment and rehabilitation.^10,22^ In this sample composed mostly of children with OI types I and IV, the children were full or community ambulators and had good PBS scores — only two children had a PBS score of <40. The ambulation level in the present study is similar to a previous study,^9^ in which 70% of the OI sample reached level 4 on the Bleck scale. Another study showed a delay in ambulation according to the disease severity, and even with the muscle weakness and lower peak force during the gait, children with OI type I could reach independent ambulation through compensations in speed and kinematics.^23^

Handgrip strength in children with OI differed significantly from the reference values of children with typical development. However, in the present study, there was no difference according to OI severity. The difference between mild and moderate/severe OI groups has already been described in a previous sample of a similar age but with a predominance of individuals with OI types III and IV; the results showed that children with more severe OI have lower grip strength than those with mild forms.^11^

There was no difference between the mild and moderate/severe groups regarding the use of bisphosphonates. A previous study showed a significant increase in muscular strength in children with OI after 36 months of treatment with bisphosphonates compared with those who did not receive the medication.^10^ Even though bisphosphonates are capable of improving physical health for a period after administration,^2^ other studies suggest that the use of bisphosphonates alone does not improve quality of life, muscular strength, or motor function.^5,24^ There was no correlation between handgrip strength and BMD, although this relationship has already been reported for a healthy population. Individuals with OI exhibit biochemical and structural alterations in bone–muscle interactions that may favor muscle weakness and bone fragility.^25,26^

The positive relationships between physical exercise, BMD, and bone health and the interaction between bone and muscle in the present study are likely negatively impacted by the low degree of physical exercise.^6,27^ The functioning level and muscular strength are correlated, as evidenced by the relationships between arm strength and the self-care domain in PEDI, and between muscular strength and the mobility domain.^9^ These effects can be explained by bone mechanics, a process by which bones adapt to external forces applied to them.^27^ However, as shown in the present study, this variable alone did not negatively impact children and adolescents enough to stop the development and performance of functional abilities or activities of daily life.^25^

Children with OI achieve high levels of independence and normal levels of social and recreational participation.^28^ There were similar results in the present study considering the PEDI-CAT daily activities domain, where only four children presented a T-score of <30, which can represent some difficulty during daily activities. Engelbert et al.^6^ evaluated children with OI with the PEDI and found a decrease in the mobility domain, especially in children with OI type III. The present study differs from that study because the sample was predominantly children with OI types I and IV. The results agree with the positive correlation between mobility and muscle strength found by Engelbert et al.,^6^ although the correlation in that study was strong, compared with moderate in the present study.

Lower mobility scores in severe types of OI have been described previously.^11^ Patients with OI type I (n=27) are most likely to have normal or close to normal PEDI scores (>80), results regarding the mobility domain. Children with OI type I have also been reported to have functional scores close to normal in the Pediatric Outcomes Data Collection Instrument (PODCI), which is another multidomain assessment tool.^23^ These two findings concur with the present study and indicate the similarities between these two groups.

The association between OI type and the mobility domain in the present study agrees with the study by Engelbert et al.^29^, in which the OI type can predict the ability to walk, at least in the household environment. In that study, the authors found that the number of fractures sustained by the children is one of the indicators of a worse prognosis for walking; the other indicator is the presence of more than two rodding procedures.^29^

Syu et al.^30^ used the Functional Independence Measure for Children (WeeFIM) in a sample of 27 children with OI. They found that children with OI types I and IV achieved full mobility and independence regarding self-care and mobility. Moreover, most children with OI, regardless of the type, achieved the full score for the cognitive domain. This finding agrees with the present study considering that only four children did not achieve a daily activities domain score in the normal range, and that 50% and 90% of the samples achieved mobility and social/cognitive domain scores in the normal range, respectively. The main limitation of the study was not achieving the proposed sample size (n=44) due to the interruption of the research because of the COVID-19 pandemic. Another limitation is that PEDI-CAT data are reported by proxy, which may lead to underestimation or overestimation in the responses.

In conclusion, children with OI present with reduced BMD and muscle strength that could impact functioning. The associated factor that mainly influences function is the number of fractures, and treatments should focus on the prevention of these events. The current findings reinforce that children with OI, especially type I, can achieve normal levels of functioning and ambulation, and this should be the focus of the rehabilitation process. This study used the PEDI-CAT, an instrument that measures abilities during real-life situations, suggesting that compensations and adaptations have been successfully developed and implemented for the activities of daily life.

## Data Availability

The database that originated the article is available with the corresponding author.
